# Transformative Frontiers: A Comprehensive Review of Emerging Technologies in Modern Healthcare

**DOI:** 10.7759/cureus.56538

**Published:** 2024-03-20

**Authors:** Sankalp Yadav

**Affiliations:** 1 Medicine, Shri Madan Lal Khurana Chest Clinic, New Delhi, IND

**Keywords:** fintech, machine learning, iomt, technology, healthcare technology, virtual reality, augmented reality (ar)

## Abstract

The rapid evolution of emerging technologies in healthcare is reshaping the field of medical practices and patient outcomes, ushering in an era of unprecedented innovation. This narrative review touches upon the transformative impacts of various technologies, including virtual reality (VR), augmented reality (AR), the internet of medical things (IoMT), remote patient monitoring (RPM), financial technology (fintech) integration, cloud migration, and the pivotal role of machine learning (ML). It emphasizes the collaborative impact of these technologies, which is reshaping the healthcare landscape. Virtual reality and AR revolutionize medical training, IoMT extends healthcare boundaries, RPM facilitates proactive care, and fintech integration enhances financial processes. Cloud migration ensures scalable and efficient data management, while ML harnesses algorithms for diagnostic precision and personalized treatment.

## Introduction and background

The rapid advancement of technology has ushered in a new era of innovation, significantly impacting various industries, and perhaps nowhere is this transformation more evident than in the field of healthcare [1]. The field of healthcare is on the cusp of a revolutionary transformation, driven by a convergence of cutting-edge technologies that promise to redefine medical practices and elevate patient care to unprecedented levels.

Among the array of emerging technologies, virtual reality (VR) and augmented reality (AR) have emerged as powerful tools with the potential to reshape medical practices and enhance patient experiences [2].

Medical professionals are increasingly turning to VR and AR for training and improving surgical skills. Virtual reality simulations provide a realistic, risk-free environment for surgeons to practice complex procedures, while AR overlays real-time data during surgeries for enhanced precision [3]. These technologies also benefit patients by providing interactive, visual representations of medical conditions, facilitating better understanding and informed decision-making about their healthcare [4]. Additionally, VR therapy is emerging as an effective treatment for mental health conditions like post-traumatic stress disorder and anxiety disorders, offering tailored immersive environments for exposure therapy [5, 6].

While the potential of VR and AR in healthcare is vast, challenges such as cost, technical complexity, and standardization hurdles must be navigated [7]. This review article aims to embark on a journey through the transformative frontiers of VR and AR, the internet of medical things (IoMT), cybersecurity measures for medical devices, remote patient monitoring (RPM), financial technology (fintech) integration, and the application of machine learning (ML) techniques within the healthcare domain.

## Review

Virtual reality and augmented reality

Both VR and AR have transcended their initial entertainment applications and are making significant inroads into the healthcare sector [8]. In the domain of medical training, VR offers an immersive learning environment that allows healthcare professionals to simulate surgeries and medical procedures. Surgeons, in particular, benefit from realistic surgical simulations that enhance their skills and provide a risk-free space to practice intricate procedures [9].

Moreover, VR is increasingly employed in patient education to enhance understanding and compliance with treatment plans. Patients can experience virtual walkthroughs of their medical conditions, visualizing the impact of diseases and potential treatments in a comprehensible manner. This not only improves patient engagement but also contributes to informed decision-making [10].

In surgical settings, AR plays a crucial role by overlaying digital information onto the surgeon's view during procedures. Real-time data visualization, such as three-dimensional (3D) reconstructions of patient anatomy, allows surgeons to navigate with unparalleled precision. This precision is especially valuable in minimally invasive surgeries, where accurate spatial awareness is critical [11].

Beyond surgical applications, VR and AR are proving beneficial in therapeutic interventions. Virtual reality therapy is being explored for treating conditions such as post-traumatic stress disorder, anxiety disorders, and phobias. The immersive nature of VR enables therapists to create controlled environments for exposure therapy, contributing to more effective and personalized mental health interventions [5, 6].

Challenges to the widespread adoption of VR and AR in healthcare include the cost of equipment, technical complexity, and the need for standardized protocols [7]. However, ongoing advancements and a growing body of evidence supporting the efficacy of these technologies underscore their potential to revolutionize medical training, patient education, and therapeutic interventions in the healthcare landscape. As technology continues to advance, VR and AR are poised to become integral tools for enhancing the skills of healthcare professionals and improving patient outcomes. Some examples are given in Table 1.

**Table 1:** Application and examples of virtual reality and augmented reality in healthcare

Application	Example 1	Example 2
Surgical training	Osso VR	Touch Surgery
Patient education	3D Organon VR Anatomy	AccuVein
Pain management	AppliedVR	EaseVRx
Mental health therapy	Limbix	Psious
Rehabilitation	MindMaze	Rehametrics
Remote consultations/Telemedicine	Proximie	Augmedix
Surgical navigation	Microsoft HoloLens	AccuVein

The internet of medical things

The IoMT represents a transformative force within the healthcare landscape, interconnecting medical devices, sensors, and systems to enhance patient care, streamline processes, and revolutionize healthcare delivery [12].

The IoMT encompasses a vast array of interconnected devices, ranging from wearable fitness trackers to advanced medical sensors. Wearable devices monitor vital signs, and physical activity, and even conduct real-time glucose monitoring, providing healthcare professionals with valuable insights into patients' health outside traditional healthcare settings. This continuous and remote monitoring fosters early detection of health issues, allowing for timely intervention and personalized treatment plans [13].

Furthermore, the IoMT facilitates the seamless exchange of data between patients and healthcare providers, contributing to a more patient-centric approach. Through interconnected health platforms and electronic health records, physicians can access real-time data, enabling more informed decision-making and personalized care strategies. Patients, in turn, gain a more active role in their healthcare management, fostering a collaborative and proactive approach to well-being [13,14].

Remote monitoring (RM) for cardiac implantable electronic devices (CIEDs) has seen increased adoption, particularly during the recent COVID-19 pandemic, offering improved healthcare accessibility and paving the way for more personalized therapy. This technology applies to various active cardiac devices like defibrillators (ICDs), pacemakers (PMs), and cardiac resynchronization therapy (CRT), either as an accessory function or a primary monitoring tool for devices like implantable loop recorders and CardioMEMS™.

Data from these devices are transmitted in real time to a database accessible to healthcare professionals, enabling prompt intervention in cases of device malfunction or detection of life-threatening arrhythmias. Patients can also initiate data transmission if they experience disturbances or suspect a deterioration in their clinical condition. Overall, RM enhances patient care by enabling continuous monitoring and timely intervention when needed.

However, the integration of IoMT is not without challenges. Privacy and security concerns surrounding the vast amounts of sensitive health data exchanged require stringent measures to safeguard patient confidentiality. Interoperability issues among diverse devices and systems also pose challenges that need to be addressed to ensure the seamless flow of information. Besides, maintaining a balance between dependence on technology and human touch is crucial for its effective use [13,14].

Looking ahead, the IoMT holds immense potential for healthcare innovation. From enhancing diagnostic capabilities through continuous monitoring to optimizing treatment plans through data-driven insights, the IoMT is at the forefront of the digital revolution in healthcare. Some examples are mentioned in Table 2.

**Table 2:** Some examples of the internet of medical things

Application	Example 1	Example 2
Remote patient monitoring	Philips Healthcare's Telehealth Solutions	Biobeat's wearable monitor
Medical device integration	Capsule Technologies' SmartLinx Solutions	Qualcomm Life's Capsule Vitals Plus
Asset management	Stanley Healthcare's AeroScout RTLS	Zebra Technologies' Zebra Savanna
Medication management	Adherium's Hailie® solution	Philips Medication Dispensing Solutions
Remote consultations/Telemedicine	Teladoc Health	Doctor on Demand
Data analytics and insights	IBM Watson Health	SAS Health Analytics

Cybersecurity measures for medical devices

While advancements in medical technology offer unprecedented benefits, the potential risks associated with inadequate cybersecurity measures cannot be understated. In an era where medical devices are becoming increasingly interconnected and reliant on digital technologies, ensuring robust cybersecurity measures is paramount. The proliferation of connected medical devices, ranging from infusion pumps to diagnostic equipment, has undeniably improved patient care and healthcare efficiency. However, this interconnectedness also exposes these devices to potential cyber threats, raising concerns about data breaches, unauthorized access, and even the potential manipulation of medical equipment [15].

According to the World Health Organization, there are more than two million different types of medical devices [16]. One of the primary challenges in medical device cybersecurity lies in the diversity of devices, each with its own specifications, software, and potential vulnerabilities. The dynamic nature of cyber threats further complicates the landscape, necessitating proactive and adaptive cybersecurity strategies [17]. Reports from the Health Information Sharing and Analysis Center, Finite State, and Securin highlighted that 64% of issues were related to software [18].

To counter these challenges, the healthcare industry is increasingly adopting comprehensive cybersecurity measures. This includes implementing robust encryption protocols, developing secure software frameworks, and establishing protocols for regular software updates and patches. Additionally, healthcare providers are investing in training programs to enhance the cybersecurity awareness of medical professionals, ensuring a collaborative approach to maintaining a secure healthcare environment [19].

Regulatory bodies and standards organizations also play a pivotal role in shaping the landscape of medical device cybersecurity. The implementation of regulations, such as the Medical Device Regulation in Europe and the pre-market and post-market guidance issued by the U.S. Food and Drug Administration, aims to establish clear guidelines for manufacturers, requiring them to prioritize cybersecurity in the design and lifecycle management of medical devices [20,21]. Some examples are mentioned in Table 3.

**Table 3:** Some examples of medical device cybersecurity measures

Cybersecurity measure	Example 1	Example 2
Encryption of data	Advanced encryption standard (AES)	Rivest-Shamir-Adleman (RSA) encryption
Authentication mechanisms	Biometric authentication (e.g., fingerprint, iris scan)	Multi-factor authentication (e.g., password, token, smart card)
Secure boot process	Ensures only trusted code is executed during startup	Hardware-based secure boot process
Regular software updates	Timely patches and updates to address security vulnerabilities	Over-the-air (OTA) updates for firmware and software updates
Intrusion detection systems (IDS)	Monitors network traffic and alerts for suspicious behavior	Host-based IDS (HIDS) monitors activities on the device itself
Access control policies	Role-based access control (RBAC)	Least privilege access principle
Device hardening	Disabling unnecessary services, ports, and functionalities	Implementing firewall rules and network segmentation measures
Secure communication protocols	Transport layer security (TLS)	Virtual private network (VPN) for secure remote access

Remote patient monitoring

Remote patient monitoring represents a paradigm shift in healthcare delivery, leveraging technology to enable continuous monitoring of patients outside traditional clinical settings [22]. It utilizes a spectrum of technologies, including wearable devices, mobile health applications, and connected medical devices, to collect and transmit patient data in real time. These technologies enable healthcare providers to remotely monitor vital signs, physiological parameters, and other relevant health metrics, providing a comprehensive and continuous view of a patient's health status [23].

One of the primary advantages of RPM is its ability to facilitate the early detection of health issues. By continuously monitoring patients with chronic conditions or those recovering from medical procedures, healthcare providers can identify subtle changes in health metrics promptly. This early detection allows for timely interventions, reducing the likelihood of complications and hospital readmissions [24].

Furthermore, RPM enhances patient engagement and empowerment. Patients can actively participate in their healthcare management by regularly tracking their health metrics and accessing personalized feedback through user-friendly interfaces. This level of engagement fosters a sense of responsibility and encourages individuals to make informed lifestyle choices, contributing to their overall well-being [25].

The integration of RPM is particularly beneficial in the management of chronic diseases such as diabetes, hypertension, and heart conditions. It allows for personalized care plans based on real-time data, optimizing treatment strategies, and improving patient outcomes [26]. Additionally, during public health emergencies or global crises, RPM becomes a valuable tool for remotely monitoring individuals, ensuring continuity of care while minimizing unnecessary exposure to healthcare facilities [27].

Despite its myriad benefits, the widespread adoption of RPM faces challenges, including issues related to reimbursement, regulatory frameworks, and ensuring equitable access to technology, especially in underserved communities [28]. Some examples are mentioned in Table 4.

**Table 4:** Some examples of remote patient monitoring (RPM)

RPM solution	Example 1	Example 2
Wearable devices	Fitbit	Apple Watch, Samsung Galaxy Watch
Remote vital sign monitors	Masimo's Radius PPG™ sensor	Biobeat's wearable monitor
Mobile health apps	Philips Healthcare's HealthSuite Digital Platform	Biofourmis' Biovitals® Platform
Telehealth platforms	Teladoc Health	Amwell Doctor on Demand
Remote monitoring Systems	Bosch Healthcare Solutions, Health Buddy Platform'	Vivify Health's remote care management platform
In-home monitoring devices	Honeywell's Genesis Touch remote patient monitoring device	ResMed's AirSense™ 10 CPAP machine with AirView™connectivity
Remote cardiac monitoring systems	BioTelemetry's ePatch™ cardiac monitoring system	Abbott's Confirm Rx™ insertable cardiac monitor

Fintech integration in healthcare

The integration of fintech into the healthcare sector marks a significant intersection of two dynamic industries, promising streamlined financial processes, enhanced security, and improved overall efficiency [29]. The traditional financial infrastructure of healthcare, often characterized by complex billing systems and delayed reimbursement processes, has been a longstanding pain point for both healthcare providers and patients [30]. The infusion of fintech solutions addresses these challenges by introducing digital innovations that optimize financial workflows, reduce administrative burdens, and contribute to a more transparent financial ecosystem [29].

One of the key areas where fintech is making a transformative impact is in payment systems. Digital payment platforms, mobile wallets, and contactless payment solutions streamline the payment process for both patients and healthcare providers. This not only expedites transactions but also enhances the overall patient experience by providing convenient and secure payment options [31].

Billing processes, notorious for their complexity and the potential for errors, are undergoing a digital transformation through fintech integration. Automated billing systems, electronic invoicing, and smart contracts facilitate accurate and efficient financial transactions, minimizing discrepancies and improving the accuracy of financial records [32].

Insurance transactions, another critical aspect of healthcare finance, are also evolving with fintech. Blockchain technology, for example, is being explored to enhance the security and transparency of insurance claims and settlements. Smart contracts on blockchain platforms can automate and streamline the claims process, reducing the likelihood of fraud and expediting the reimbursement cycle [33].

Despite these advancements, the integration of fintech in healthcare finance is not without challenges. Concerns related to data security, privacy, and regulatory compliance require careful consideration. Additionally, ensuring equal access to digital financial services and addressing the digital divide is crucial to preventing disparities in healthcare finance [34]. Some examples are mentioned in Table 5.

**Table 5:** Some examples of financial technology (fintech) integration in healthcare

Fintech integration in healthcare	Example 1	Example 2
Healthcare payment solutions	Waystar	InstaMed
Health savings accounts (HSAs)	Lively	Further
Medical financing platforms	CareCredit	Prosper Healthcare Lending
Healthcare insurance platforms	Oscar Health	Bright Health
Healthcare expense management	Alegeus	HealthEquity
Healthcare billing automation	Cedar	Patientco
Medical crowdfunding platforms	GoFundMe Medical	FundRazr Medical

Cloud migration in healthcare

The healthcare industry is experiencing a transformative shift with the adoption of cloud computing technologies [35]. Traditionally, healthcare organizations managed vast amounts of patient data through on-premise servers, posing challenges related to scalability, accessibility, and data security. Cloud migration addresses these challenges by providing a scalable and secure environment for storing, managing, and sharing healthcare data.

One of the primary advantages of cloud migration in healthcare is the enhanced scalability of storage resources. Cloud-based platforms allow healthcare organizations to dynamically scale their storage capacity based on demand, accommodating the exponential growth of medical data generated from electronic health records (EHRs), medical imaging, and genomic data [36].

Accessibility and collaboration are pivotal aspects of modern healthcare delivery, and cloud computing facilitates seamless access to patient data from various locations. Healthcare providers can securely access patient records, diagnostic images, and treatment plans from any authorized device with an internet connection, promoting efficient and collaborative care [37].

The cloud also supports advanced data analytics and machine learning applications, allowing healthcare organizations to derive meaningful insights from large datasets. This is particularly valuable for personalized medicine, population health management, and predictive analytics, contributing to more informed decision-making and improved patient outcomes [38].

While the benefits of cloud migration are substantial, challenges such as data privacy, security, and regulatory compliance must be carefully addressed. Healthcare providers must ensure that cloud service providers adhere to stringent security standards, implement encryption protocols, and comply with healthcare data protection regulations, such as the Health Insurance Portability and Accountability Act (HIPAA) in the United States [37]. Some examples are mentioned in Table 6.

**Table 6:** Some examples of cloud migration in healthcare

Cloud migration solutions in healthcare	Example 1	Example 2
Electronic health record (EHR) systems	Epic Systems Corporation	Cerner Corporation
Picture archiving and communication systems (PACS)	Ambra Health	Life Image
Health information exchange (HIE) platforms	Redox	Health Gorilla
Telehealth platforms	Teladoc Health	Amwell
Healthcare analytics platforms	Google Cloud Healthcare	IBM Watson Health
Medical imaging analysis tools	Aidoc	Zebra Medical Vision
Remote patient monitoring platforms	Biofourmis	Philips Healthcare
Health information management (HIM)	Mirth Corporation (NextGen)	Hyland Healthcare (OnBase)

Machine learning techniques in healthcare

Machine learning techniques have emerged as powerful tools in healthcare, promising to revolutionize diagnostics, treatment plans, and overall patient care. At its core, ML involves the development of algorithms that enable computers to learn patterns and make predictions or decisions without explicit programming. In healthcare, this translates to the ability to analyze vast datasets, identify complex patterns, and derive meaningful insights to inform medical decision-making [38].

One of the primary applications of ML in healthcare is in diagnostics. Machine learning algorithms can analyze medical imaging data, such as X-rays, magnetic resonance imaging, and computed tomography scans, with remarkable accuracy. Automated image recognition and classification help healthcare professionals detect abnormalities, tumors, and other conditions at an early stage, facilitating prompt intervention and treatment [39].

Beyond diagnostics, ML contributes to the personalization of treatment plans. By analyzing patient data, including genetic information, medical history, and lifestyle factors, ML algorithms can assist in tailoring treatment strategies to individual patients. This approach, known as precision medicine, aims to maximize treatment efficacy while minimizing adverse effects [40].

Predictive analytics is another key area where ML excels. By analyzing historical patient data, ML algorithms can predict disease progression, potential complications, and patient outcomes. This information empowers healthcare providers to implement proactive measures, such as preventive interventions and personalized care plans, contributing to improved patient care and resource allocation [41].

Despite the promising applications, ML in healthcare is not without challenges. Ethical considerations, data privacy, and the interpretability of complex algorithms are among the critical issues that need to be addressed. Ensuring transparency and establishing trust in ML models are essential for their successful integration into clinical practice [42]. Some examples are mentioned in Table 7.

**Table 7:** Some examples of machine learning techniques in healthcare

Technique/Application	Example 1	Example 2
Medical imaging analysis	Deep learning for MRI image interpretation	Google's DeepMind Health's artificial intelligence (AI)-based retinal disease detection
Clinical decision support systems	IBM Watson Health's Clinical Decision Support System	Ada Health's AI-driven symptom checker
Predictive analytics	Predicting patient readmissions based on electronic health records	Identifying sepsis in ICU patients using vital signs and lab values
Drug discovery and development	Atomwise's AI-driven drug discovery platform	BenevolentAI's AI platform for drug discovery and development
Personalized medicine	Genomic sequencing and machine learning for personalized treatment plans	IBM Watson for Genomics for cancer treatment
Healthcare fraud detection	Detecting fraudulent insurance claims using anomaly detection techniques	Optum's AI-based fraud detection system
Natural language processing	Extracting information from clinical notes and reports	Linguamatics' NLP platform for mining unstructured healthcare data

The exploration of emerging technologies in healthcare unveils a landscape of unprecedented innovation, promising transformative impacts on medical practices and patient outcomes. From the immersive experiences of VR and AR to the interconnected networks of the IoMT, each technology reshapes the healthcare paradigm. These technologies redefine medical training, providing healthcare professionals with advanced tools for skill development and offering patients immersive educational experiences. The IoMT extends healthcare beyond traditional boundaries, enabling continuous monitoring and personalized interventions, thereby advancing patient-centered care. Robust cybersecurity measures for medical devices are critical to securing the integrity of patient data and ensuring the trustworthiness of healthcare technologies. Remote patient monitoring emerges as a linchpin for proactive healthcare, allowing for early interventions and empowering individuals to manage their health remotely. Fintech integration and cloud migration optimize financial processes and data storage, offering efficiency gains and accessibility. Machine learning stands out as a transformative force, offering insights from vast datasets for diagnostics, treatment personalization, and predictive analytics. Despite the challenges, the potential for personalized medicine and data-driven decision-making heralds a new era in healthcare. As these technologies advance, the healthcare industry is on the brink of a transformative era. Ethical considerations, privacy concerns, and security challenges must be addressed to ensure the equitable distribution of benefits and the preservation of patient-centered care. By fostering a culture of responsible innovation, the healthcare community can unlock the full potential of emerging technologies, leading to a future where healthcare is not only technologically advanced but also ethically sound and patient-centric.

## Conclusions

In conclusion, the domain of healthcare is undergoing a profound and transformative evolution fueled by emerging technologies. The multifaceted impacts of these technologies are reshaping the way medical practices are conducted and influencing patient outcomes in unprecedented ways. As the healthcare industry stands on the brink of this transformative era, it is crucial to acknowledge and address ethical considerations, privacy concerns, and security challenges. Responsible innovation is paramount to ensuring the equitable distribution of benefits and the preservation of patient-centered care. By cultivating a culture of ethical and responsible use of these technologies, the healthcare community can realize its full potential, leading to a future where healthcare is not only technologically advanced but also ethically sound and truly patient-centered.
